# Colorectal cancer cell-derived extracellular vesicles induce phenotypic alteration of T cells into tumor-growth supporting cells with transforming growth factor-β1-mediated suppression

**DOI:** 10.18632/oncotarget.7041

**Published:** 2016-01-27

**Authors:** Nami Yamada, Yuki Kuranaga, Minami Kumazaki, Haruka Shinohara, Kohei Taniguchi, Yukihiro Akao

**Affiliations:** ^1^ United Graduate School of Drug Discovery and Medical Information Sciences, Gifu University, Gifu, Japan

**Keywords:** colorectal cancer, exosome, extracellular vesicle, TGF-β1, tumor-stromal interaction

## Abstract

Emerging studies on tumor cell-derived extracellular vesicles (EVs) have shown the biological significance in tumor development and microenvironment through reprogramming immune cells around cancer cells. In this study, we used colorectal cancer cells as EVs donor, and T cells as recipients to examine whether EVs impair the T cell function. As a result, we found that colorectal cancer cell-derived EVs (CRC-EVs) were enriched with TGF-β1. Interestingly, CRC-EVs induced phenotypic alteration of the T cells to Treg-like cells through activating TGF-β/Smad signaling and inactivating SAPK signaling. Furthermore, the CRC-EVs-induced-Treg-like cells had a remarkable tumor-growth promoting activity *in vitro* and *in vivo*. These results suggest that colorectal cancer cells utilize EVs to tame immune cells for their prosperity.

## INTRODUCTION

Cancer cell-derived extracellular vesicles (EVs), which carries DNAs, proteins, mRNAs and microRNAs, have been intensively studied recently as a novel microenvironment modulators and potential cancer biomarkers [1, 2]. EVs are membranous vesicular structures classified into 2 main classes of these vesicles: shed microvesicles (SMVs) and exosomes. SMVs are 100-400 nm membranous vesicles derived from outward blebbing of various kinds of cell surfaces upon a variety of types of stimulation. Exosomes are smaller vesicles (50-100 nm in diameter) released by exocytosis of multivesicular bodies (MVBs) [2, 3]. Currently available purification methods cannot fully discriminate between SMVs and exosomes; therefore, we use a collective term “extracellular vesicles” (EVs) for all the extracellular vesicles between 50 and 400 nm in diameter. It is considered that contents of EVs vary depending on the cell type of origin and so do their function within the tumor microenvironment. However, a precise role of EVs still needed to be validated.

Transforming growth factor-β1 (TGF-β1) is a multipotent cytokine involved in the regulation of proliferation, differentiation and survival of various kinds of cells, and those effects depend on cellular contexts [4]. In immune system, TGF-β1 is required for the induction of CD4^+^Foxp3^+^ T cells called regulatory T cells (Tregs). Tregs were highly enriched in immune suppressor activities and responsible for maintaining the homeostasis of immune system [5]. In many malignant tumors, increased Tregs population in tumor microenvironment or within the peripheral blood of patients indicates a state of tumor immunotolerance and that is known as a poor prognosis marker [6]. Accumulated Tregs in tumor microenvironment suppress immune response against tumor cells by inducing apoptosis of T cells through secretion of perforin and granzyme, inhibiting maturation of antigen presenting cells (APCs) *via* cytotoxic T lymphocyte antigen-4 (CTLA-4) and lymphocyte-activation gene 3 (LAG3), or producing immune suppressive cytokines such as IL-10 [7]. Therefore, management of Tregs’ accumulation in tumor microenvironment has been focused as a potent anti-cancer strategy [8].

In this study, we examined to determine how colorectal cancer cells communicate with T cells *via* EVs. Here, we show that colorectal cancer cell-derived EVs (CRC-EVs) suppress the proliferation of T cells through the perturbation of intracellular signalings including MAPK, AKT and TGF-β/Smad signaling. Consequently CRC-EVs induce phenotypic alteration of the T cells into tumor-growth promoting cells having several characteristics of Tregs. These findings can be an important clue to understand intercellular communication between tumor cells and immune cells *via* EVs that contribute to the tumor progression.

## RESULTS

### Colorectal cancer cells secreted TGF-β1 *via* EVs

**Figure 1:**
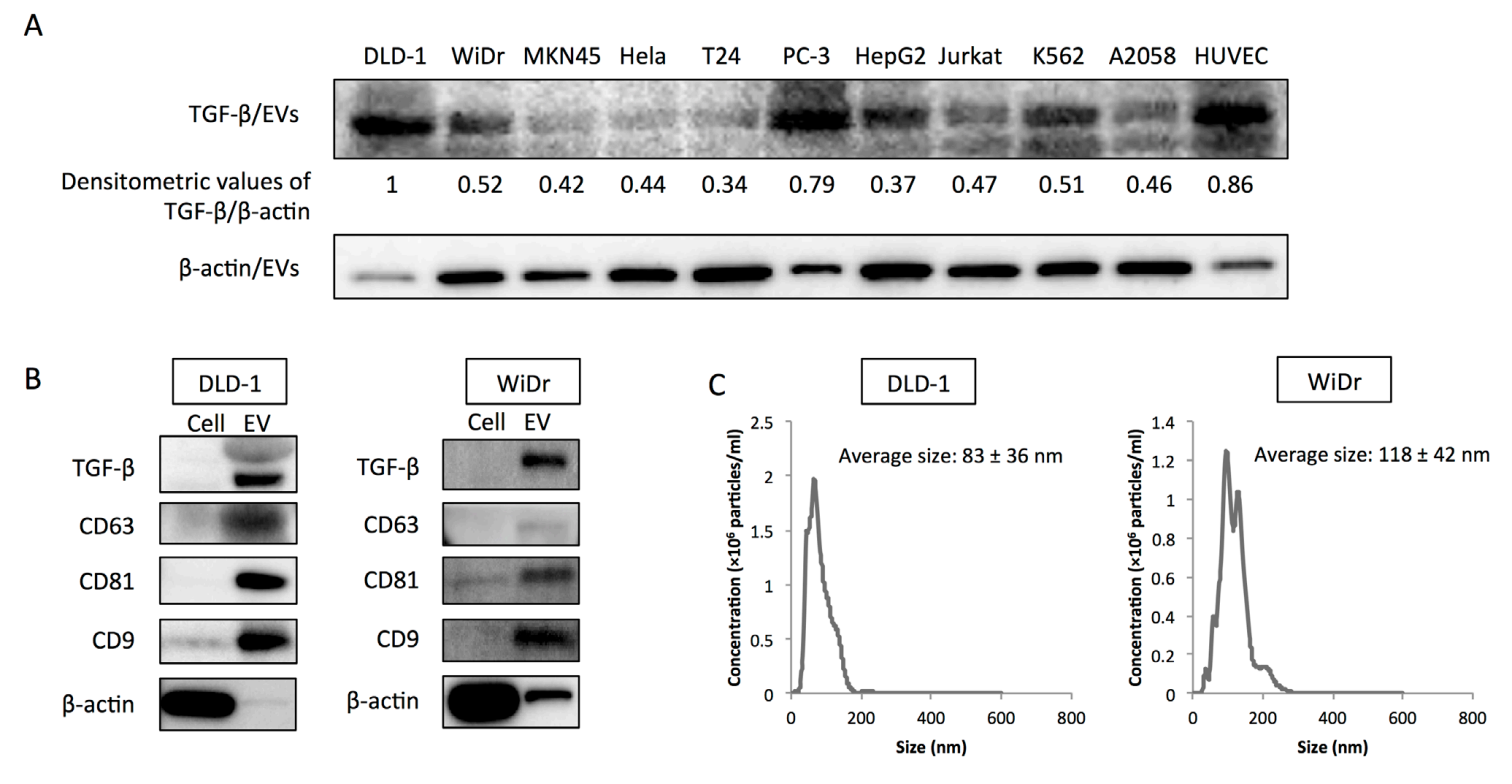
TGF-β1 is secreted *via* extracellular vesicles (EVs) by colorectal cancer cells **A.** Relative expression levels of secreted TGF-β1 via EVs (TGF-β/EVs) in various cell lines. Densitometric values are shown for TGF-β1 protein. β-actin was used as a normalizer. The relative expression level of TGF-β/EVs in DLD-1 cells was taken as “1”. **B.** Protein expression profiles of EVs from DLD-1 and WiDr cells. CD63, CD81, and CD9 are well-known identification markers for EVs. **C.** Size distribution of colorectal cancer cell-derived EVs (CRC-EVs) analyzed by NanoSight (nanoparticle tracking system). Average sizes of CRC-EVs are shown as the mean ± SD.

In a previous study, we found that colorectal cancer cells secreted TGF-β1 *via* EVs [9]. To examine the specificity of TGF-β1 secretion by colorectal cancer cells, secreted TGF-β1 levels in various cell lines were evaluated (Figure 1A). DLD-1 and WiDr cells secreted relatively higher level of TGF-β1 *via* EVs compared with those of other cell lines, except PC-3 cells and HUVECs. To further examine the purity of EVs, protein expression profiles of the EVs derived from DLD-1, WiDr and PC-3 cells were examined. CD63, CD81, and CD9 are often used as identification markers for EVs. Whereas β-actin was barely detected in EVs, these identification markers were dominantly expressed in EVs compared with those in their paired cells (Figure 1B, and Supplementary Figure 1A). The EVs were also enriched in TGF-β1 compared with the cells, indicating that colorectal cancer cells and PC-3 cells positively secreted TGF-β1 into their surrounding environment. Furthermore, NTA revealed that colorectal cancer cells and PC-3 cells secreted a heterogeneous population of EVs in size (average size in DLD-1: 83 ± 36 nm; average size in WiDr: 118 ± 42 nm, and average size in PC-3: 129 ± 61 nm) (Figure 1C, and Supplementary Figure 1B). Given the identification of specific proteins and size distribution detected by NTA, we concluded that our purification method efficiently yielded “EVs”, a mixture of exosomes and SMVs.

### CRC-EVs containing TGF-β1 inhibited cell growth of Jurkat cells

**Figure 2:**
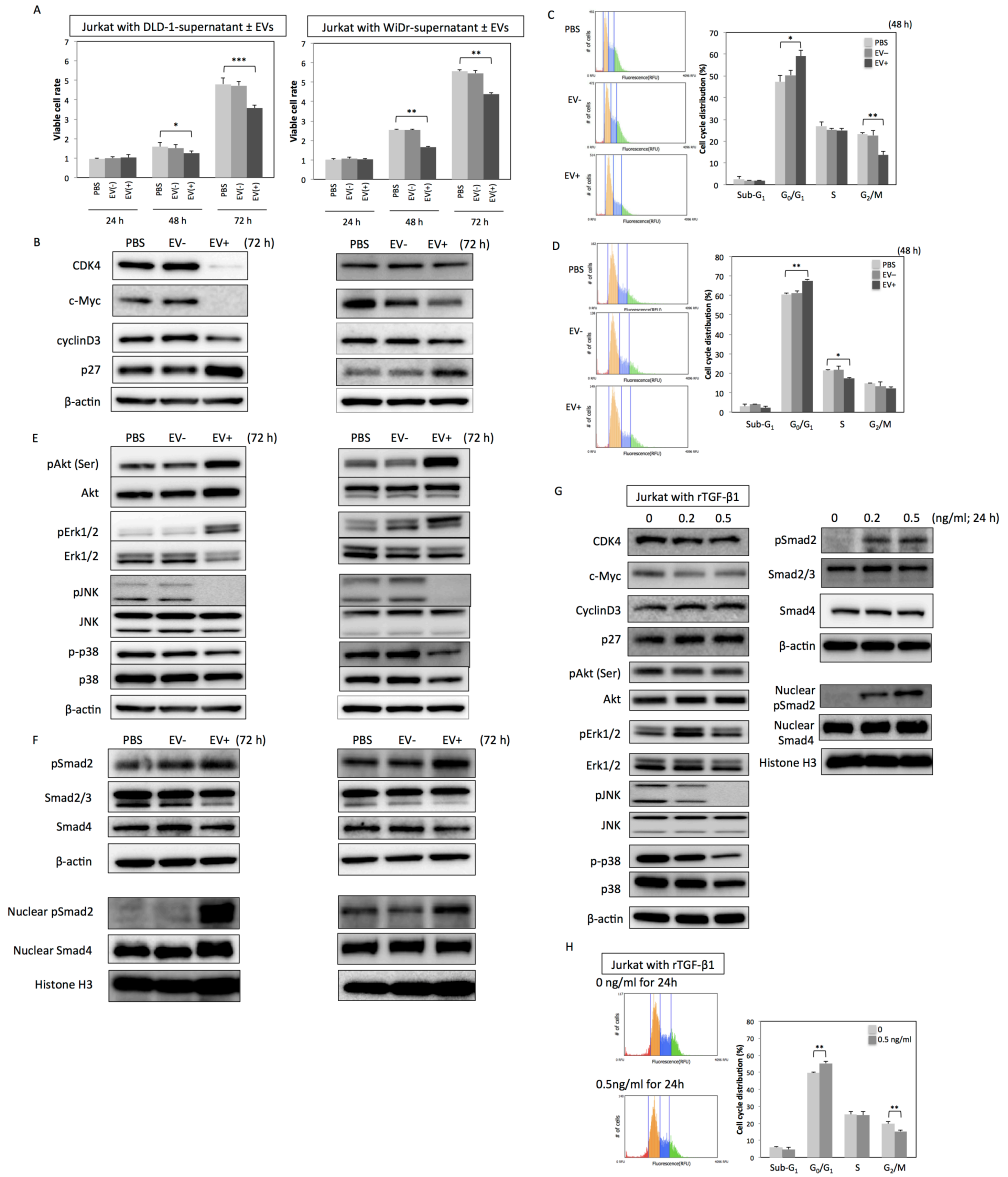
CRC-EVs inhibit proliferation of Jurkat cells through EV-associated TGF-β1 **A.** Cell viability of Jurkat cells incubated with PBS, CRC-supernatant deprived of EVs [EV (−)], or CRC-supernatant with collected EVs [EV (+)] at 24, 48, and 72 h. **B.** Expression profiles of cell cycle-related proteins at 72 h after incubation with PBS, EV (−), or EV (+). **C.**, **D.** Cell cycle distribution in Jurkat cells analyzed by cytometer at 48 h after incubation with PBS, EV (−), or EV (+) derived from DLD-1 cells **C.** and WiDr cells **D.**. **E.**, **F.** Expression profiles of kinase cascades-related proteins **E.** and Smad signaling-related proteins **F.** in Jurkat cells at 72h after incubation with PBS, EV (−), or EV (+). **G.**, **H.** Protein expression profiles **G.** and cell cycle distribution **H.** in Jurkat cells at 24 h after treatment with recombinant TGF-β1 (rTGF-β1) at a concentration of 0 - 0.5 ng/ml. The *P* values in “A,” “C,” “D,” and “H” are indicated as follow: *P < 0.05, **P < 0.01, and ***P < 0.001.

Next, to validate the biological roles of the CRC-EVs containing TGF-β1, Jurkat cells as T-like cells were incubated with CRC-EVs. Incubation with the EVs significantly decreased the number of viable Jurkat cells (Figure 2A), down-regulated the expression of cell-cycle promoter proteins such as CDK4, c-Myc, CyclinD3, and up-regulated cell-cycle inhibitor protein p27 (Figure 2B). Same inhibitory effects on the cell viability and cell-cycle-related proteins were also observed in Jurkat cells incubated with PC-3 cell-derived EVs (Supplementary Figure 1C and 1D). Analysis of cell-cycle distribution by using cytometer also revealed the G_0_/G_1_ arrest of Jurkat cells (Figure 2C and 2D). Sub-G_1_ phase, which mostly indicates apoptotic cell population, was unchanged (Figure 2C and 2D). Furthermore, while JNK and p38 (SAPKs) were inactivated by the incubation with CRC-EVs, AKT and Erk1/2 were activated (Figure 2E). However, in Jurkat cells incubated with PC-3 cell-derived EVs, JNK, AKT, and Erk1/2 were inactivated but p38 status was unchanged (Supplementary Figure 1D). Activation of Smad signaling was observed in both Jurkat cells incubated with CRC-EVs and PC-3 cell-derived EVs (Figure 2F and Supplementary Figure 1E). Treatment with recombinant TGF-β1 (rTGF-β1) reproduced the results obtained in Jurkat cells incubated with CRC-EVs (Figure 2G and 2H). Taken together, these results indicate that CRC-EVs attenuate proliferation of the recipient Jurkat cells via EV-associated TGF-β1 (TGF-β/EVs).

### CRC-EVs containing TGF-β1 up-regulated Treg-related genes in Jurkat cells, PBMCs and T cells

**Figure 3:**
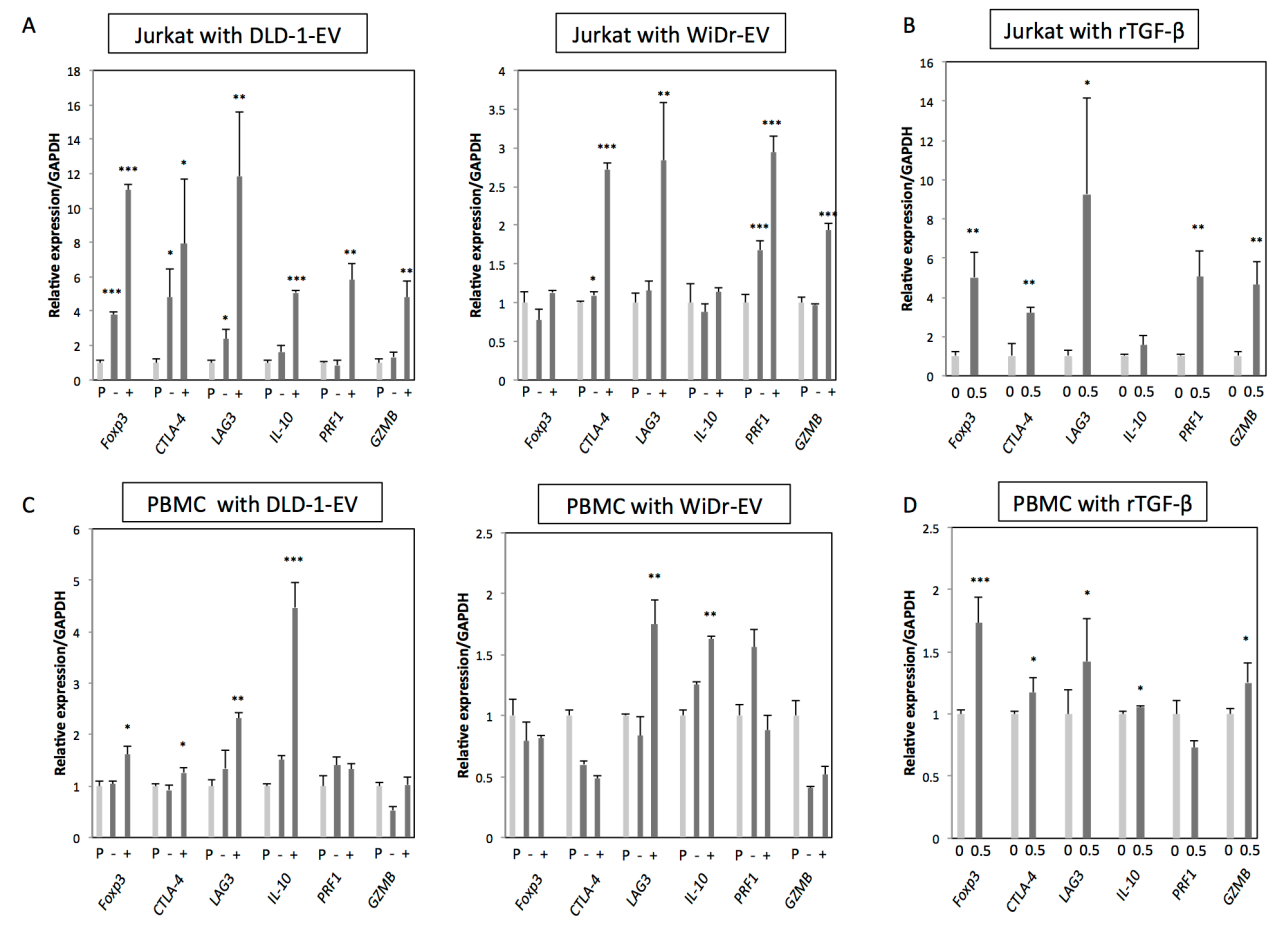

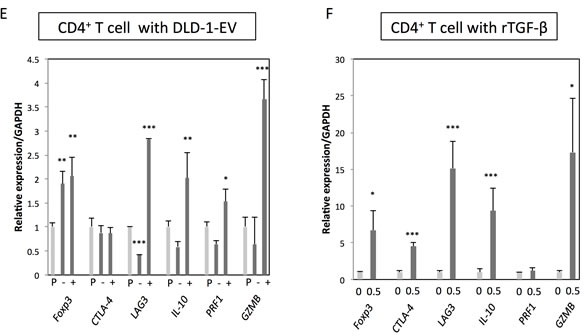
CRC-EVs up-regulate Treg-related genes in Jurkat cells, PBMCs and CD4+ T cells **A**.-**F**. Expression profiles of Treg-related genes at 48 h after incubation with PBS, EV (−), or EV (+), and at 24 h after treatment with rTGF-β1 at a concentration of 0 - 0.5 ng/ml in Jurkat cells **A**.,**B**., PBMCs **C**., **D**. and CD4^+^ T cells **E**., **F**. The *P* values are indicated as follow: *P < 0.05, **P < 0.01, and ***P < 0.001.

Next, to further validate the biological function of TGF-β/EVs, the resultant phenotypes induced by incubation with CRC-EVs or treatment with rTGF-β1 were examined in Jurkat cells. *FoxP3, CTLA-4, LAG3, IL-10, PRF1*, and *GZMB* are well-known Treg-related genes, which are often up-regulated in Tregs induced by activated TGF-β/Smad signaling. As shown in Figure 3A, all Treg-related genes were significantly up-regulated in Jurkat cells treated with DLD-1 cell-derived EVs. Compared with that, WiDr cell-derived EVs mildly up-regulated Treg-related genes except *FoxP3* and *IL-10* (Figure 3A). Treatment with rTGF-β1 also up-regulated these genes except *IL-10* (Figure 3B). These results suggest that CRC-EVs containing TGF-β1 can alter the phenotype of recipient Jurkat cells to Treg-like cells. We also used PBMCs and peripheral blood CD4^+^ T cells to validate that the activation of TGF-β/Smad signaling was not limited to Jurkat cells. As shown in Figure 3C and 3E, DLD-1 cell-derived EVs up-regulated *FoxP3, CTLA-4, LAG3, and IL-10* in PBMCs and *FoxP3, LAG3, IL-10, PRF1*, and *GZMB* in CD4^+^ T cells. However, WiDr cell-derived EVs up-regulated only *LAG3* and *IL-10* in PBMCs (Figure 3C). Treatment with rTGF-β1 also up-regulated these genes except *PRF1* in PBMCs (Figure 3D) and CD4^+^ T cells (Figure 3F). Additionally, PC-3 cell-derived EVs also up-regulated *FoxP3, LAG3*, and *IL-10* in Jurkat cells (Supplementary Figure 1E).

### Up-regulation of Treg-related genes by CRC-EVs was mainly due to the TGF-β1/EVs

**Figure 4:**
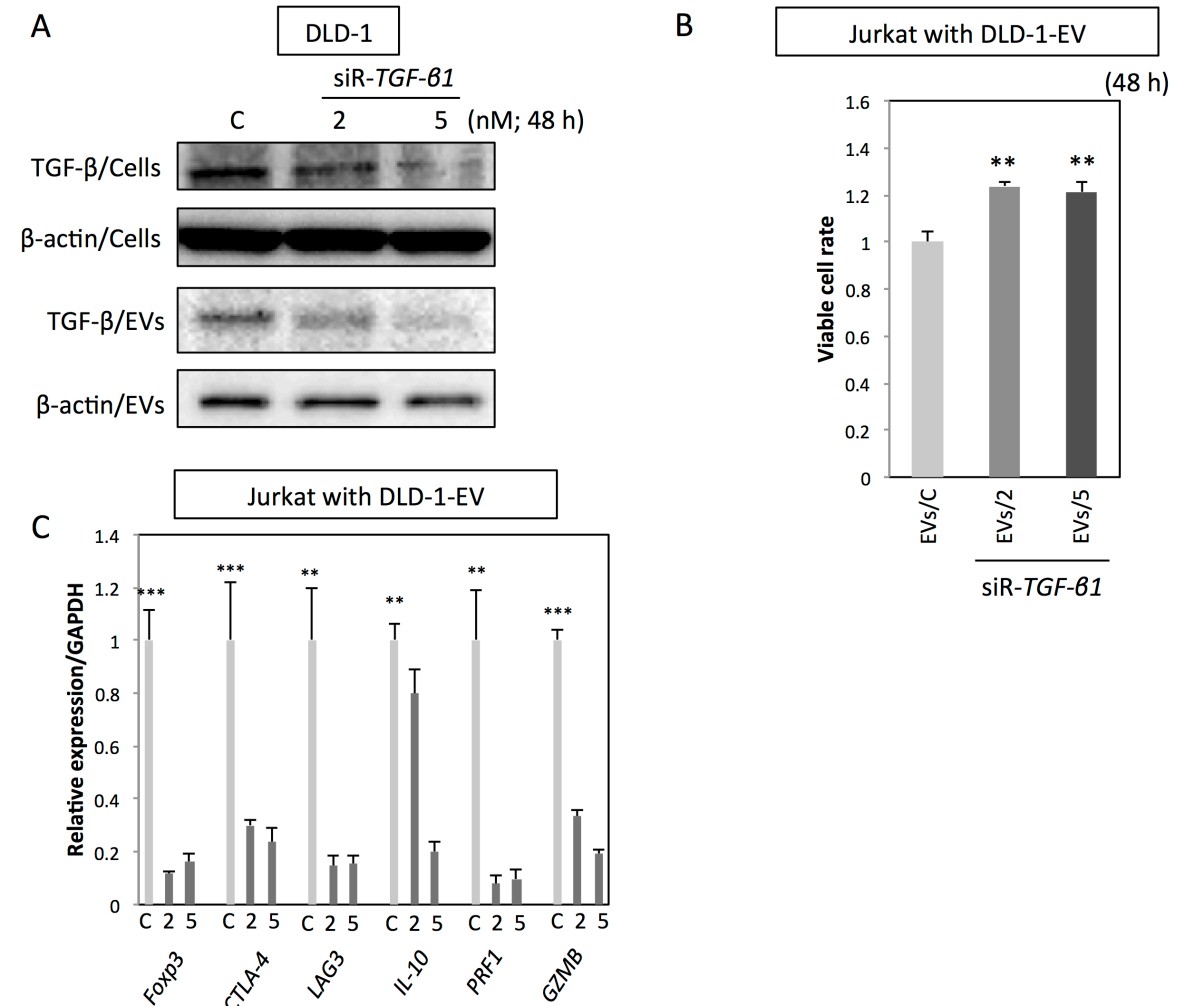
Up-regulation of Treg-related genes by CRC-EVs is mainly due to the EV-associated TGF-β1 **A.** Intracellular TGF-β1 (TGF-β/Cells) and secreted TGF-β1 (TGF-β/EVs) in DLD-1 cells at 48 h after transfection with siR-*TGF-β1* (2 and 5 nM). (B, C) Cell viability **B.** and expression profiles of Treg-related genes **C.** in Jurkat cells at 48 h after incubation with EVs derived from DLD-1 cells transfected with siR-*TGF-β1* [EVs/C; EVs from control siRNA transfected-DLD-1 cells, EVs/2; EVs from siR-*TGF-β* (2 nM) transfected-DLD-1 cells, and EVs/5; EVs from siR-*TGF-β* (5 nM) transfected-DLD-1 cells]. The *P* values in “B” and “C” are indicated as follow: *P < 0.05, **P < 0.01, and ***P < 0.001.

**Figure 5:**
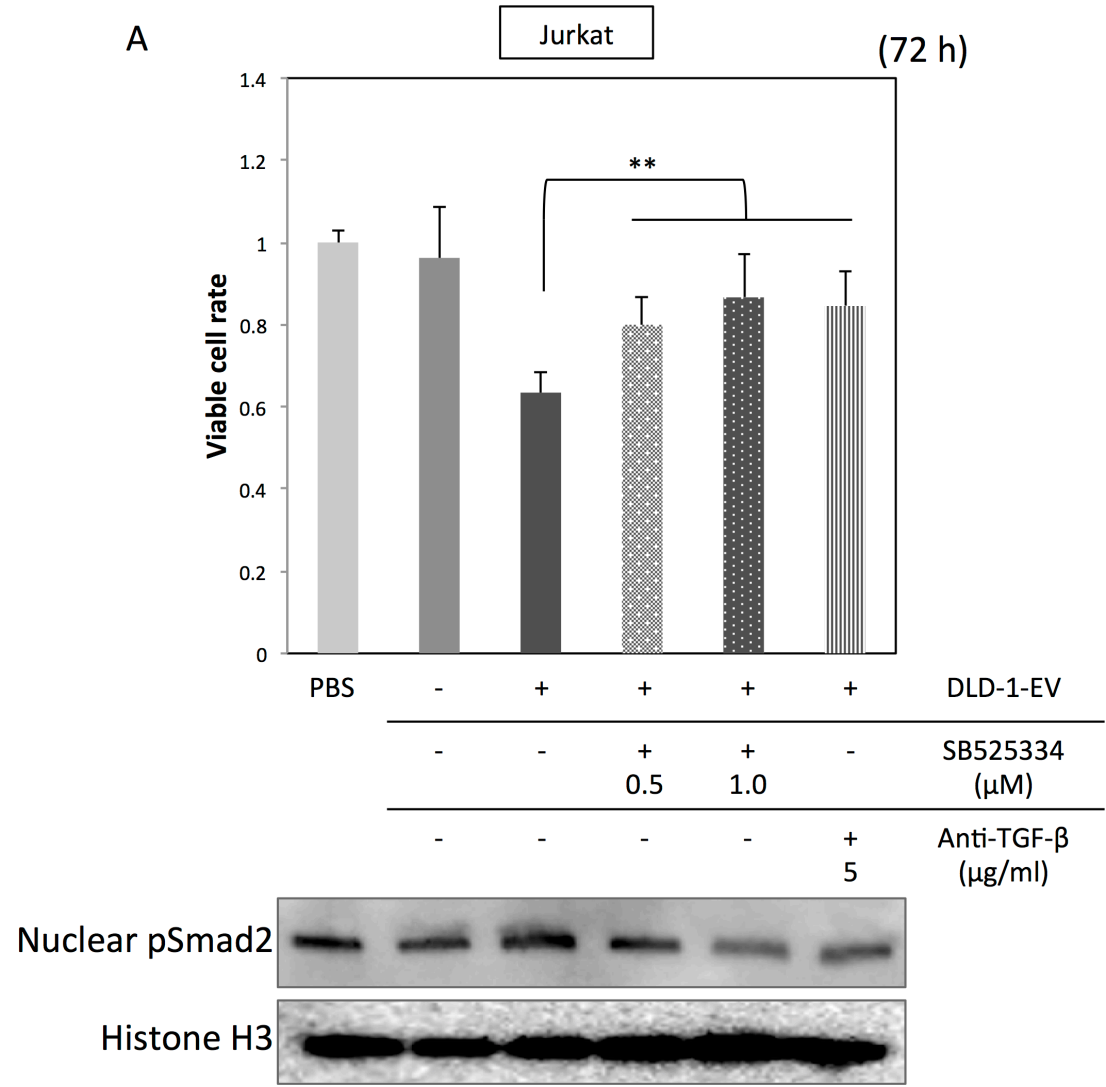

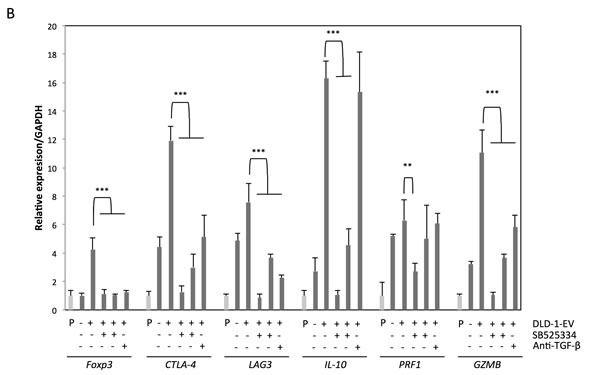
Treatment with TGF-β1 antagonist or inhibitor of TGF-β receptor I diminishes the effects of CRC-EVs on Jurkat cells (A, B) Cell viability and activation status of Smad signaling **A.**, and expression profiles of Treg-related genes **B.** in Jurkat cells incubated with PBS, EV (−), EV (+), EV (+) with SB525334 (0.5-1.0 μM) or EV (+) with anti-TGF-β1 neutralizing antibody (5 μg/ml). The *P* values in “A” and “B” are indicated as follow: *P < 0.05, **P < 0.01, and ***P < 0.001.

To further confirm the specific roles of TGF-β/EVs in inductive function of Treg-related genes, DLD-1 cells were transfected with siR-*TGF-β1*, and their EVs were isolated for the incubation with Jurkat cells. As shown in Figure 4A, transfection with siR-*TGF-β1* significantly down-regulated both intracellular and secreted levels of TGF-β1 in DLD-1 cells. EVs secreted by siR-*TGF-β1*-transfected-DLD-1 cells (EVs/2 and EVs/5) had attenuated effects on Jurkat cells compared with that of EVs secreted by control-siRNA-transfected-DLD-1 cells (EVs/C) (Figure 4B and 4C). Furthermore, treatment with TGF-β1 antagonist or inhibitor of TGF-β receptor I diminished the effects of CRC-EVs on cell viability, Smad signaling (Figure 5A) and expression levels of Treg-related genes (Figure 5B) in Jurkat cells. These results suggest that up-regulation of Treg-related genes by EVs was mainly due to the secreted TGF-β1 *via* EVs.

### EV-treated Jurkat cells contributed to tumor progression

**Figure 6:**
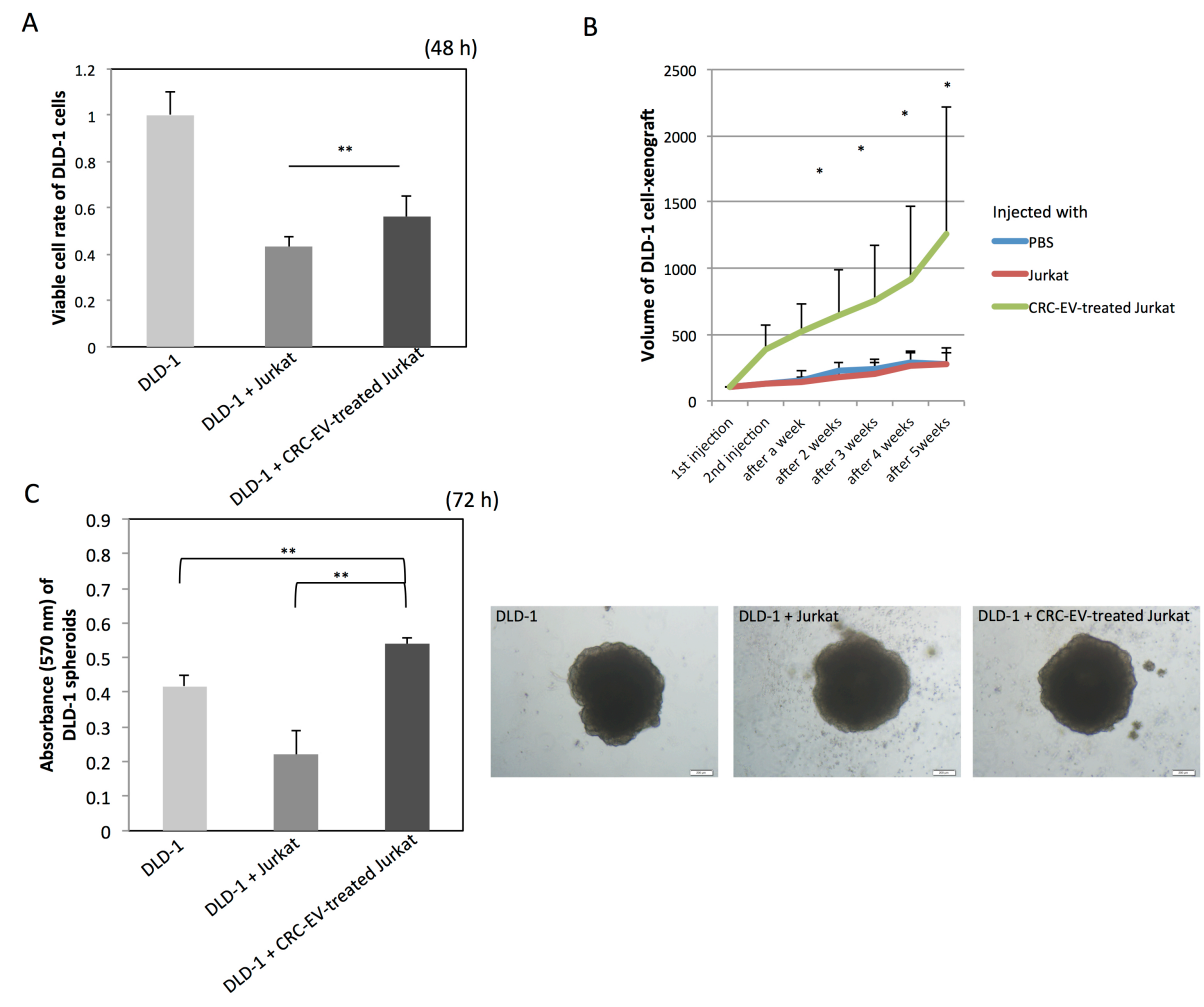
CRC-EV-treated Jurkat cells promote tumor progression **A.** Cell viability of DLD-1 cells co-cultured with Jurkat cells or CRC-EV-treated Jurkat cells for 48 h in a well with a partition of the 4 μm porous filter. **B.** Tumor volumes of DLD-1 cell-xenografted mice injected with PBS, Jurkat cells, or CRC-EV-treated Jurkat cells around the tumors. **C.** Viability of DLD-1 cell-derived spheroids co-cultured with Jurkat cells or CRC-EV-treated Jurkat cells for 72 h evaluated by the MTT assay. The *P* values in “A”, “B” and “C” are indicated as follow: *P < 0.05 and **P < 0.01. Jurkat cells were incubated with CRC-EVs for 48 h before the co-culture with DLD-1 cells **A.**, spheroids **C.** or the injection **B**.

To examine the function of the CRC-EV-treated Jurkat cells, DLD-1 cells were co-cultured with the Jurkat cells in a well with a partition of the 4 μm porous filter (Figure 6A). Compared with DLD-1 cells cultured with Jurkat cells, the cell proliferation of the DLD-1 cells co-cultured with the CRC-EV-treated Jurkat cells was significantly promoted at 48 h. Like the result of the *in vitro* experiment, tumor progression was significantly promoted in the mice injected with the CRC-EV-treated Jurkat cells (Figure 6B). Furthermore, the 3D spheroid colorimetric viability assay also showed that the CRC-EV-treated Jurkat cells significantly promoted expansion of DLD-1 cell-derived spheroids (Figure 6C). These results indicate that the CRC-EV-treated Jurkat cells acted as tumor-growth promoting cells *in vitro* and *in vivo*. However, Jurkat cells treated with rTGF-β1 or incubated with PC-3 cell-derived EVs did not show such a tumor-growth promoting activity on DLD-1 cells *in vitro* (Supplementary Figure 1F).

## DISCUSSION

The present study revealed that colorectal cancer cells secrete EVs to inhibit immune response against themselves by inhibiting T cell proliferation, and altering phenotype of T cells into tumor-growth promoting cells, more useful cells for them. We first considered that the CRC-EV-treated Jurkat cells were differentiated into Tregs, as already reported in several studies [10, 11]. Indeed, expression levels of several Treg-related mRNAs were increased in the CRC-EV-treated Jurkat cells, PBMCs and T cells. However, the CRC-EV-treated Jurkat cells had a remarkable tumor-promoting activity both *in vitro* and *in vivo*. Additionally, the tumor-promoting effect of CRC-EV-treated Jurkat cells was observed both with and without cell-to-cell contact. Those were not considered to be a general function of Tregs. The mechanism of the remarkable tumor-growth induced by the CRC-EV-treated Jurkat cells remains unclear in this study. However, it is considered that the activation of AKT and ERK signalings, and inactivation of JNK and p38 signalings should contribute to the tumor-promoting activity of the CRC-EV-treated Jurkat cells. JNK and p38 signalings are known as the pro-cell death signaling. Therefore, the inactivation of these signalings is considered to be a compensatory response for the arrested cell-cycle to prevent polarization to cell death and support differentiation. The rTGF-β1 treatment inactivated JNK and p38 signalings in Jurkat cells similar to CRC-EVs. PC-3-EVs also inactivated JNK signaling but not p38 signaling. Based on these results, it is considered that inactivation of SAPK signaling in Jurkat cells is mainly due to TGF-β1. However, the activation status of AKT and ERK signalings was varied; CRC-EVs activated AKT and ERK signaling in Jurkat cells, however, the rTGF-β1 treatment did not. PC-3-EVs, on the contrary, inactivated AKT and ERK signalings. These results indicate that AKT and ERK signalings are activated by some genetic products of EVs other than TGF-β1 (cargo X). Furthermore, only CRC-EVs could confer a tumor-growth promoting phenotype upon T-reg like Jurkat cells. Therefore, it is considered that the tumor-growth promoting function is due to the cargo X in CRC-EVs, which activated AKT and ERK signalings in Treg-like Jurkat cells. Further studies on the cargo X will be needed.

Previously we validated that CRC-EVs promote angiogenesis through up-regulating Smad 1/5/8 signaling in the recipient endothelial cells [9]. In that study, TGF-β1 and miR-1246 contained within the CRC-EVs contribute to the pro-angiogenesis. Altogether, it is considered that cancer cell-derived EVs have a consequential purpose to contribute to the tumor growth even if the recipient cells varied. Our goal is to elucidate the role of cancer-cell derived EVs in the tumor microenvironment. Our results suggest that cancer cells utilize EVs to modulate functions of their surrounding cells to maintain and expand themselves. Preventing EV-secretion by tumor cells and/or removing tumor cell-derived EVs could be a potential therapeutic strategy and a worthy of further study.

## MATERIALS AND METHODS

### Cell culture and cell viability

Human colon cancer DLD-1 and WiDr cells, and human T-cell leukemia Jurkat cells (JCRB cell bank, Osaka, Japan) were cultured in RPMI-1640 medium supplemented with 10% (vol/vol) heat-inactivated FBS (Sigma-Aldrich, St Louis, MO). Peripheral blood CD4^+^ T cells were obtained from Lonza (Walkersville, MD) and cultured in LGM-3 medium supplemented with 10% (vol/vol) heat-inactivated FBS. CRC-EVs were incubated with Jurkat cells, PBMCs or CD4^+^ T cells at a concentration of 0.5 × 10^18^ - 1 × 10^18^ particles/ 0.5 × 10^5^ cells. CRC-EV-treated Jurkat cells were obtained by the incubation with Jurkat cells and CRC-EVs for 48 h. Recombinant TGF-β1 (Life Technologies, Grand Island, NY) was used at a concentration of 0.2 - 0.5 ng/ml. Inhibitor of TGF-β receptor I (SB525334; Selleck Chemicals, Houston, TX) was used at a concentration of 0.5 - 1 μM. DLD-1 cells and Jurkat cells were co-cultured in the RPMI medium mentioned above at a concentration of 0.5 × 10^5^ cells/ml each. DLD-1 cells were seeded in the wells, and Jurkat cells were seeded in the chamber inserts using the 4 μm porous Biocoat Matrigel chamber insert (BD Biosciences, San Jose, CA).

### Peripheral blood mononuclear cells (PBMCs) purification

Human peripheral blood samples were obtained from healthy donors with informed consent of the subjects and following ethical approval. PBMCs were purified by density gradient centrifugation using Ficoll-Paque PLUS (GE Healthcare Bio-Sciences, Uppsala, Sweden). PBMC were resuspended at 10^6^ cells/ml in RPMI-1640, the same medium mentioned above.

### Quantitative RT-PCR using real-time PCR

Total RNA was isolated from cultured cells by using a NucleoSpin miRNA isolation kit (TaKaRa, Otsu, Japan). For determination of the expression levels of *Forkhead box P3 (FoxP3), Cytotoxic T lymphocyte angtigen-4 (CTLA-4), Lymphocyte-activation gene 3 (LAG3), IL-10, Perforin-1 (PRF1), Granzyme B (GZMB)* and *GAPDH* mRNAs, total RNA was reverse-transcribed with a PrimeScript^®^ RT Reagent Kit (TaKaRa, Otsu, Japan). Real-time PCR was then performed with primers specific for them by using THUNDERBIRD SYBR qPCR Mix (TOYOBO, Osaka, Japan). The primers for *FoxP3, CTLA-4, LAG3, IL-10, PRF1, GZMB* and *GAPDH* were as follow: *FoxP3*-sense, 5′-TCCAGGACAGGCCACATTTC-3′; *FoxP3*-antisense, 5′-GACACCATTTGCCAGCAGTG-3′; *CTLA-4*-sense, 5′-AGGTGACTGAAGTCTGTGCG-3′; *CTLA-4*-antisense, 5′-CATGAGCTCCACCTTGCAGA-3′; *LAG3*-sense, 5′-GATGGCTTCAACGTCTCCAT-3′; *LAG3*-antisense, 5′-CTTGGCAGTGAGGAAAGACC-3′; *IL-10*-sense, 5′-AAGACCCAGACATCAAGGCG-3′; *IL-10*-antisense, 5′-AGGCATTCTTCACCTGCTCC-3′; *PRF1*-sense, 5′-TCCTAAGCCCACCAGCAATG-3′; *PRF1*-antisense, 5′-AAGGAGGCCGTCATCTTGTG-3′; *GZMB*-sense, 5′-CAGCTGGAGAGAAAGGCCAA-3′; *GZMB*-antisense, 5′-TGGCGTAAGTCAGATTCGCA-3′; *GAPDH*-sense, 5′-CTCAGACGGCAGGTCAGGTCCACC-3′; *GAPDH*-antisense, 5′-CCACCCATGGCAAATTCCATGGCA-3′. The relative expression levels were calculated by the ΔΔC_t_ method. *GAPDH* was used as an endogenous normalizer for *FoxP3, CTLA-4, LAG3, IL-10, PRF1* and *GZMB* expressions.

### Isolation of colorectal cancer cell-derived extracellular vesicles (CRC-EVs)

For the collection of CRC-EVs, RPMI medium supplemented with EV-deprived FBS was made as follows: FBS was centrifuged at 3,000 rpm for 5 min, and its supernatant was filtered through a Millex-HV Filter Unit (0.45-μm pores). The flow-through fraction was ultracentrifuged at 100,000 rpm for 3 hours. Without disturbing the EV pellet, the supernatant was carefully removed and used as EV-deprived FBS, which was then added to the RPMI medium (EV-deprived RPMI). DLD-1 and WiDr cells (1 × 10^6^ each) were cultured for 96 hours at 37°C in dishes containing 40 ml of EV-deprived RPMI. The culture medium was then collected and centrifuged at 2,000 rpm for 5 min. The resulting supernatant was filtered through the 0.45-μm-pore-filter to remove cell debris and then through the 0.22-μm-pore-filter (a Millex-GV Filter Unit 0.22 μm pores; Merck Millipore) to remove the large EVs and/or apoptotic bodies. The flow-through fraction was ultracentrifuged at 100,000 rpm for 3 hours. Without disturbing the EV pellet, 1 ml of supernatant was carefully removed and used as colorectal cancer cell-supernatant without EVs (CRC-supernatant deprived of EVs). Another 1 ml was taken and used to resuspend the EV pellet (CRC-supernatant with collected EVs). For analysis of the miRNA expression profile of CRC-EVs, RNA lysis buffer supplied in a NucleoSpin miRNA isolation kit (TaKaRa) was added to an EV pellet (obtained as described above); and RNA samples were prepared. To analyze protein expression profile of CRC-EVs, protein lysis buffer described below was added to another EV pellet; and protein samples were prepared. Anti-TGF-β1 neutralizing antibody (R&D Systems, Minneapolis, MN) was used at a concentration of 5 μg/ml.

### Nanoparticle tracking analysis (NTA)

Nanoparticle tracking analysis (NTA) is a method used for detecting secreted EVs in a liquid sample. CRC-EVs suspended in 1 ml of PBS were analyzed by using an NTA Version 2.3 Build 0034 instrument (NanoSight, Wiltshire, UK). Samples were diluted at 1:1,000 in PBS and analyzed.

### Western blotting

Protein extraction and Western blotting experiments were performed as described previously [12]. Primary antibodies used were as follow: anti-TGF-β1, CDK4, c-Myc, CyclinD3, p27, phospho-Akt, Akt, phospho-Erk1/2, Erk1/2, phospho-JNK, JNK, phospho-p38, p38, phospho-Smad 2, Smad 2/3, Smad 4, and Histone H3 (Cell Signaling, Danvers, MA); anti-CD63, CD81, and CD9 (Santa Cruz, Santa Cruz, CA); and anti-β-actin (Sigma-Aldrich). HRP-conjugated horse anti-mouse and goat anti-rabbit IgG (Cell Signaling) were used as secondary antibodies.

### Cell cycle analysis

Quantification of cellular DNA content at 48 h after treatment with CRC-EVs was determined by using a cytometer. Briefly, the cells were harvested and fixed with 70% cold ethanol at -20°C overnight. The fixed cells were washed twice with PBS, resuspended in 100 μl PBS-based propidium iodide solution containing 0.1% Triton X-100 (Wako, Osaka, Japan), 0.2 mg/ml RNase A (Life Technologies), and 20 μg/ml propidium iodide (Life Technologies), and incubated for 30 min at room temperature protected from the light. The DNA content in the cells was analyzed through the cytometer (The Tail^®^ Image-Based Cytometer, Life Technologies).

### Transfection with short-interfering RNA for TGF-β1

DLD-1 cells were seeded at a concentration of 0.5 × 10^5^ cells/ml (10-30% confluence) on the day before the transfection. Short-interfering RNA (siRNA) for *TGF-β1* (siR-*TGF-β1*; Invitrogen, Carlsbad, CA) was used for the transfection of the cells, which was achieved by using cationic liposomes, Lipofectamine RNAiMAX (Invitrogen), according to the manufacturer's protocol. The nonspecific control siRNA (HSS, Hokkaido, Japan) sequence was 5′-GUAGGAGUAGUGAAAGGCC-3′ [13, 14]. The sequence of siR-*TGF-β1* were 5′-CAGCACGTGGAGCTGTACCAGAAAT-3′ and 5′-TGGGCACTGTTGAAGTGCCTTACAT-3′. The effects manifested by the introduction of siR-*TGF-β1* into the cells were assessed at selected time points after the transfection.

### Human tumor xenograft model

Our institute's committee for ethics in animal experimentation approved all animal experimental protocols. Animal experiments were conducted in accordance with the guidelines for Animal Experiments of Gifu International Institute of Biotechnology. Six female athymic nude mice were inoculated into their subcutaneous flanks (each side of flank per mouse) with DLD-1 cells at 2 × 10^6^ cells in 100 μl of PBS, and the inoculation time was set as day 0. From day 10, control group (*n* = 2) received PBS injections around the tumors twice a week for 2 weeks. Another group (*n* = 2) received injections of Jurkat cells (1 × 10^5^ cells in 100 μl of PBS) around the tumors. The other group (*n* = 2) received injections of EV-treated Jurkat cells (1 × 10^5^ cells in 100 μl of PBS) around the tumors. Jurkat cells and EV-treated Jurkat cells were irradiated with a 3 Gy electron beam prior to the injections. At day 24, the mice were sacrificed by an intraperitoneal injection of 50 mg/kg pentobarbital sodium (Somnopentyl; Kyoritsu Seiyaku Co., Tokyo, Japan). Tumor size were monitored by measuring the length (*L*), width (*W*), and depth (*D*), and the volumes were estimated according to the following formula: *V* (mm^3^) = *L × W × D ×* 0.5.

### The 3D Spheroid colorimetric proliferation/viability assay

The 3D spheroid colorimetric proliferation/viability assay was performed according to the manufacturer's protocol of a Cultrex 3-D spheroid Colorimetric Proliferation/Viability Assay Reagent Kit (Trevigen, Inc., Gaithersburg MD). Briefly, DLD-1 cells were suspended in Spheroid Formation ECM at a concentration of 3,000 cells/50 μl, and seeded in the 3D Culture Qualified 96 Well Spheroid Formation Plate at 50 μl/well. The plate was centrifuged at 200 × g for 3 min at room temperature and incubated at 37°C for 72 h to promote spheroid formation. Then, Irradiated Jurkat cells or EV-treated Jurkat cells (0.5 × 10^4^ cells respectively) were added to the culture media of the DLD-1 cell-derived spheroids and co-cultured for 72 h. The spheroids were photographed with the microscope (CKX41, Olympus, Tokyo, Japan), and analyzed images using image analysis software to measure changes in the area of the structures to determine the extent of 3-D culture spheroid expansion for each sample. After that, MTT assay was also performed according to the manufacturer's protocol. Absorbance was read at 570 nm by the microplate reader (iMark microplate reader, Bio-Rad Laboratories Inc., Hercules, CA).

### Statistics

Each examination was performed in triplicate. Statistical analyses were performed by using GraphPad Prism software system (GraphPad Software, Inc., La Jolla, CA). Statistical significances of differences were evaluated by performing the two-sided Student's *t*-test. The values were represented as the mean ± standard deviation. A *p* value < 0.05 was considered to be statistically significant.

## SUPPLEMENTARY MATERIALS FIGURE


