# Supplementary material for: Colorectal cancer cell-derived extracellular vesicles induce phenotypic alteration of T cells into tumor-growth supporting cells with transforming growth factor-β1-mediated suppression
Source: Oncotarget. 2016 Jan 27;7(19):27033–43. doi: 10.18632/oncotarget.7041 (PMC5053630; doi:10.18632/oncotarget.7041)
Supplement: Supplementary file 1 [file oncotarget-07-27033-s001.pdf]

Colorectal cancer cell-derived extracellular vesicles induce phenotypic alteration of T cells into tumor-growth supporting cells with transforming growth factor-β1-mediated suppression

Supplementary Material

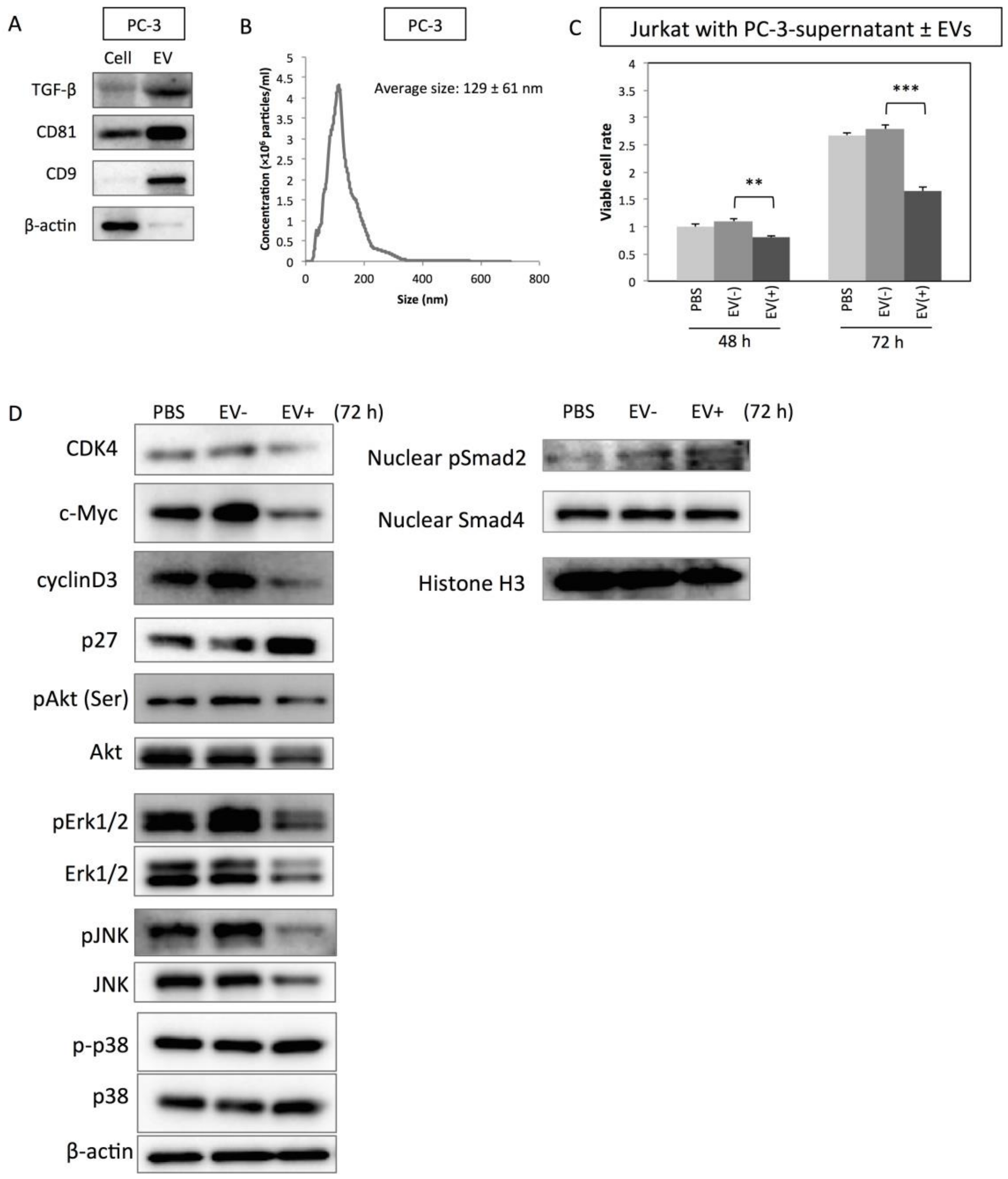

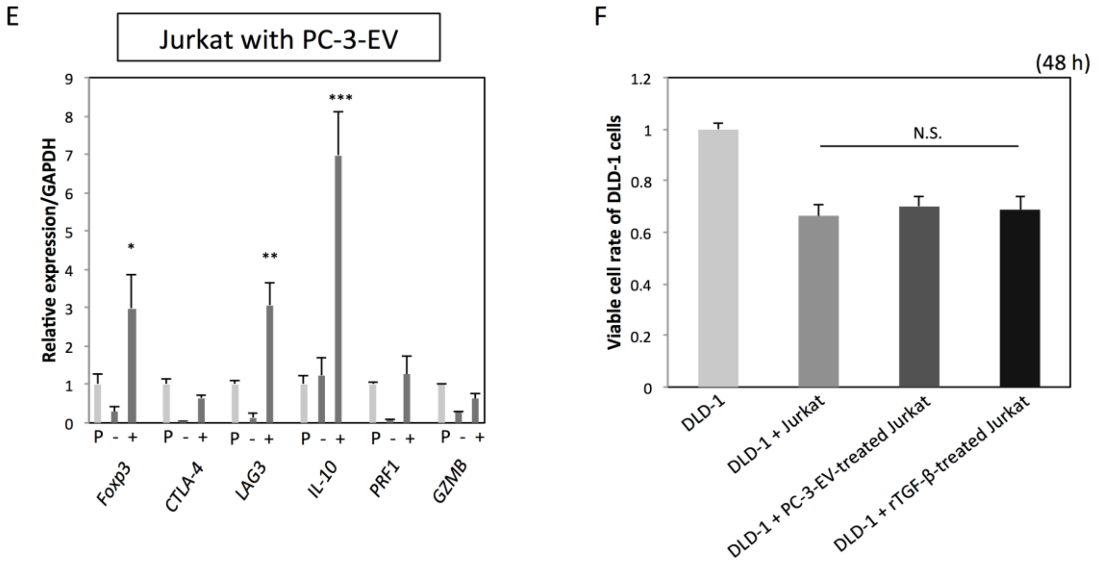

Supplementary Figure 1. The effects of EVs derived from PC-3 cells on Jurkat cells. (A) Protein expression profiles of EVs from PC-3 cells. (B) Size distribution of PC-3 cell-derived EVs (PC-3-EVs) analyzed by NanoSight (nanoparticle tracking system). Average sizes of PC-3-EVs are shown as the mean ± SD. (C) Cell viability of Jurkat cells incubated with PBS, PC-3-supernatant deprived of EVs [EV (-)], or PC-3-supernatant with collected EVs [EV (+)] at 48 and 72 h. (D) Expression profiles of kinase cascades-related proteins and Smad signaling-related proteins in Jurkat cells at 72h after incubation with PBS, EV (-), or EV (+). (E) Expression profiles of Treg-related genes in Jurkat cells at 48 h after incubation with PBS, EV (-), or EV (+). (F) Cell viability of DLD-1 cells co-cultured with Jurkat cells, PC-3-EV-treated Jurkat cells, or rTGF-β-treated Jurkat cells for 48 h in a well with a partition of the 4 μm porous filter. Jurkat cells were incubated with PC-3-EVs for 48 h or treated with rTGF-β1 (0.5 ng/ml) for 24 h before the co-culture with DLD-1 cells. The *P* values in “C”, “E” and “F” are indicated as follow: N.S. Not significant, *$P < 0.05$ and **$P < 0.01$, and ***$P < 0.001$.
